# Metal-Organic Framework Membranes and Membrane Reactors: Versatile Separations and Intensified Processes

**DOI:** 10.34133/2020/1583451

**Published:** 2020-05-12

**Authors:** Yujie Ban, Na Cao, Weishen Yang

**Affiliations:** ^1^State Key Laboratory of Catalysis, Dalian Institute of Chemical Physics, Chinese Academy of Sciences, 457 Zhongshan Road, Dalian 116023, China; ^2^University of Chinese Academy of Sciences, 19A Yuquan Road, Beijing 100039, China

## Abstract

Metal-organic frameworks are an emerging and fascinating category of porous solids that can be self-assembled with metal-based cations linked by organic molecules. The unique features of MOFs in porosity (or surface areas), together with their diversity for chemical components and architectures, make MOFs attractive candidates in many applications. MOF membranes represent a long-term endeavor to convert MOF crystals in the lab to potentially industry-available commodities, which, as a promising alternative to distillation, provide a bright future for energy-efficient separation technologies closely related with chemicals, the environment, and energy. The membrane reactor shows a typical intensified process strategy by combining the catalytic reaction with the membrane separation in one unit. This review highlights the recent process of MOF-based membranes and the importance of MOF-based membrane reactors in relative intensified chemical processes.

## 1. Introduction

Metal-organic frameworks (MOFs) are one of the most rapid growing categories of porous solids in past decades. MOFs have infinite crystalline lattices that are composed of inorganic building units (metal-based cations) coordinated with organic molecules. The diversity of both inorganic and organic moieties gives rise to thousands of interesting structures that display large internal surface areas (the highest reported is 7,000m^2^g^−1^) [1], ultralow densities, and uniformly arranged cavities and portals of molecular dimensions. MOF materials show enormous potential in many territories including catalysis, gas storage, and drug delivery. Gas- and liquid-phase separations based on MOFs have been the most widely examined application. The possibility of MOFs for tailorable component characteristics and porous architectures makes these materials attractive in the separation territory.

A separation membrane represents a thin and selective barrier, which comprises firmly connected micro-/nanocrystals in the MOF case. An ideal MOF membrane possesses well-aligned nanopores to extend in a two-dimensional (2D) plane, acting as “molecular gates” to permit the transportation of target molecules. Engineering of the MOF membrane is an important step forward to transform laboratory-synthesized MOF crystals to potentially industry-viable products for separations, which are emerging as a promising alternative to distillation in terms of energy consumption. The past few years have witnessed the remarkable growth and breakthroughs of MOF membranes in both gas and liquid separations.

**Figure 1:**
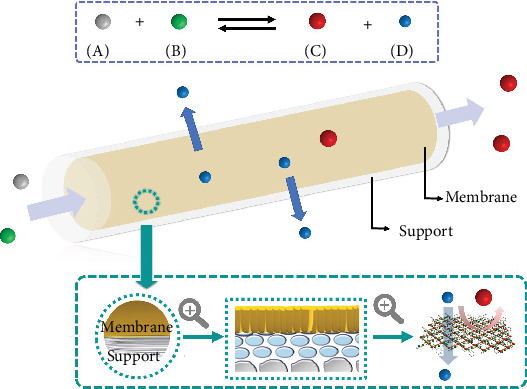
Conceptional illustration of a tubular membrane reactor applied in an intensified process.

Integrating a chemical reaction (usually catalytic conversion) with a concurrently efficient separation technology manifests a typical intensified process strategy to realize sustainable growth in energy consumption and chemical production and simultaneously relieve pressure in the environment. The membrane reactor (MR) appears to be such a molecular “super factory” that can simultaneously realize chemical conversion and membrane separation (Figure 1). The benefits of using MRs compared with conventional reactor are [2] (i) economy efficiency as a result of a compact and integrated unit and (ii) high productivity and selectivity because of shifting equilibrium.

In this review, we firstly focus on versatile MOF membranes from the past few years, including H_2_-, CO_2_-, alkene-, water-, and organic-selective MOF membranes from fundamental synthesis to application. Then, we highlight three types of MRs, namely, H_2_ production MRs, dehydration MRs, and organic extract MRs, and relative intensified processes, which are closely related with chemical, petrochemical, and biochemical production, environmental remediation, and the energy sector.

## 2. MOF Membranes: Versatile Separations at Molecular Dimensions

### 2.1. H_2_-Selective Membranes

H_2_ is a clean energy source. In recent years, demands for H_2_ have been growing continuously. H_2_-based gases always contain various coproducts, by-products, and residual reactants such as CO_2_, CO, CH_4_, and H_2_O. Molecular sieving MOF membranes with an aperture size comparable to molecular diameters have been demonstrated to be one of the most promising candidates that are suitable for splitting smaller H_2_ molecules from the larger ones.

**Figure 2:**
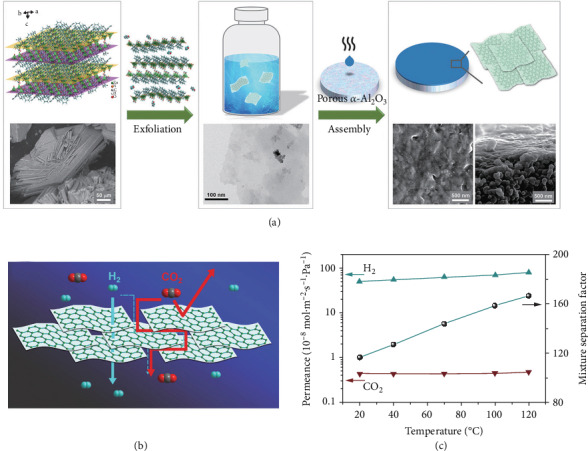
(a) Representation of the top-down method from the precursor exfoliation to nanosheet assembly. (b) Illustration of transportation pathways through the nanosheet membrane. (c) The H_2_/CO_2_ separation performances of the nanosheet membrane with the elevated operating temperature. Reproduced from Ref. [4] with permission from Wiley, copyright 2017.

Two-dimensional (2D) Zn_2_(Bim)_4_ nanosheet membranes developed by Yang and coworkers represented a typical membrane with extremely narrow aperture windows (~0.21 nm) applied in H_2_/CO_2_ separation. The top-down synthesis method for the membranes included the exfoliation of lamellar MOF materials, followed by the reassembly of the nanosheets into crack-free membranes [3]. We also present another ultrathin nanosheet membrane, namely, the Zn_2_(Bim)_3_ membrane with bilayered nanosheets (~1.6 nm) as building blocks (Figure 2). One single Zn_2_(Bim)_3_ nanosheet has amphiprotic nature in theory, since benzimidazolate ligands are coordinated with zinc nodes on one side of the nanosheet and water molecules are dangling bonded on the other side. In membrane separation, H_2_ passed through the aperture windows while CO_2_ was detained in the interlayer space of the nanosheet membranes, thus leading to an excellent separation performance at elevated temperature, which holds promise in actual H_2_ production and CO_2_ capture [4]. Zhao and coworkers also synthesized nm thick MOF membranes [Ni_8_ (5-bbdc)_6_(m-OH)_4_)] composed of well-aligned exfoliated MOF nanosheets, displaying a reversed thermoswitchable molecular sieving characteristic for H_2_/CO_2_ separation [5]. In addition to the top-down method, Zhang and coworkers proposed a controllable and environment-friendly synthesis manner for H_2_-selective ultrathin Co_2_(Bim)_4_ membranes, that is, the solvent-free ligand vapor phase transformation (VPT) [6]. The Co-containing precursor layer on the substrate directly converted to membranes with altered thickness of around 57-750 nm.

Besides, the development of a chemical hybridization strategy motivated the development of MOF-based hybrid membranes, such as MOF@MOF, MOF@GO (graphene oxide, GO), MOF@g-C_3_N_4_ (2D graphitic carbon nitride, g-C_3_N_4_), MOF@COF (covalent organic framework, COF), and MOF@LDH (layered double hydroxides, LDH) [7–11]. The synergistic sieving effect of the MOF-based hybrid membrane demonstrated an unprecedented H_2_ separation performance, lighting on the tailored synthesis of membranes with diversified nanostructures for highly efficient separation.

### 2.2. CO_2_-Selective Membranes

Anthropogenic emissions of greenhouse gases in particular for CO_2_ result in global warming. To address climate change issues, there is growing interest in seeking commercially viable opportunities for CO_2_ capture. In addition, CO_2_ is an impurity in natural gas, biogas, and other gas steams. An amine solution can absorb CO_2_ by reaction in a high-volume scale, but this process is energy intensive because heat must be applied to remove CO_2_ for amine regeneration. Developing membrane technologies for the CO_2_ sequestration and separation that have much smaller energy footprints is of great significance to both energy and the environment.

**Figure 3:**
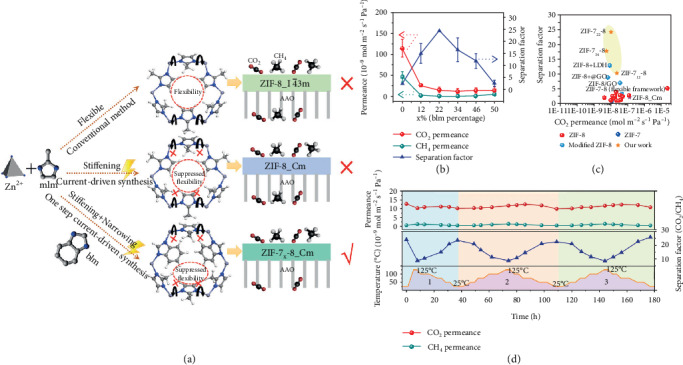
(a) The concept of stiffened hybrid-linker ZIF membranes. (b) Separation characteristics of the hybrid-linker ZIF membranes. (c) Summary of performances of ZIF membranes in literature. (d) Stability of the current membrane when splitting CO_2_ from CH_4_. Reproduced from Ref. [16] with permission from Wiley, copyright 2019.

MOF materials enable an efficient membrane separation as a result of the unprecedented CO_2_-philicity of MOFs even in the presence of moisture. However, it still poses a great challenge for MOF membranes to efficiently separate gas mixtures by distinguishing the minor differences in molecular size because of linker mobility of MOFs that permits much larger molecules to pass through the aperture windows. That is why ZIF-8 (also called MAF-4 [12]) membranes that attract extensive interest have been demonstrated to have poor CO_2_/CH_4_ selectivity (~2.5) [13–15]. Wang and coworkers rapidly constructed 500 nm thin dual-linker ZIF-7_22_-8 membrane in an electrochemical reactor (~20 min). Owing to the inherent stiffened framework of ZIF-8 formed in the electric field and tailoring the pores of ZIF-8 via a hybrid-linker method, the obtained ZIF-7_22_-8 showed sharp molecular sieving properties for CO_2_/CH_4_, exceeding many other ZIF-8 or modified ZIF-8 membranes (Figure 3). The 180 h temperature swing permeations confirmed the stability of ZIF-7_22_-8 membranes in relative aggressive environments [16].

In addition to the pure MOF membrane, another feasible membrane solution for CO_2_ capture tends to favor the use of mixed matrix membranes (MMMs) by combining the processability of polymers and the permeable and selective characteristics of MOF fillers, improving the separation benchmark and surpassing Robeson's upper limit of the pristine polymer membranes [17–19]. Yang and coworkers quantitatively studied the synergistically adsorptive and diffusive effect of MOF fillers by using a classical solution-diffusion gas transportation model, indicating that MOF fillers (ZIF-8) performed as a molecular sieving pathway with good adsorption affinity to CO_2_ molecules for achieving highly efficient CO_2_/CH_4_ separation [20]. The tailoring of the pore structure and chemical components of MOFs can be anticipated to improve the separation benchmark of MMMs by the tuning of the molecularly exclusive and CO_2_-philic characteristics. We proposed cage-occupying concept to tailor the dimension of the SOD cage and molecular recognition of ZIF-8. Ionic liquid (IL) as the cage occupants was directly confined into the SOD cage of ZIF-8 during synthesis of ZIF-8 in IL medium, reducing the effective cage size of ZIF-8 to be between CO_2_ and N_2_. The cut-off windows accordingly shifted from six-membered aperture rings to the effective blocked cages. The gas selectivity of MMMs that were decorated with IL@ZIF-8 was remarkably enhanced when sieving CO_2_/CH_4_ and CO_2_/N_2_ [21]. Dispersion of amine-functionalized UiO-66 into a polymer membrane also led to a substantial enhancement of CO_2_ selectivity as a result of the interaction of -NH_2_ with CO_2_ [22]. Simultaneously, it is imperative to regulate the chemical interaction of MOFs with the polymer matrix through fine-tuning of the crystal size, micro-/nanostructure, and surface functionality of MOFs (also functionality of polymers) [23–26].

### 2.3. Alkene-Selective Membranes

Alkenes (e.g., ethene, propylene, butadiene, and butene isomers) are critical platform compounds in petrochemical industries. Alkenes are mixed with alkanes in cracking products of hydrocarbon feedstocks. Alkene/alkane separations (e.g., separation of ethene/ethane and propylene/propane) are commonly implemented on a massive scale by distillation, which are among the most difficult and costly separation processes because these two chemicals have very similar boiling points. Membranes as an alternative to distillation are urgently needed to make alkene/alkane separation more energy efficient.

**Figure 4:**
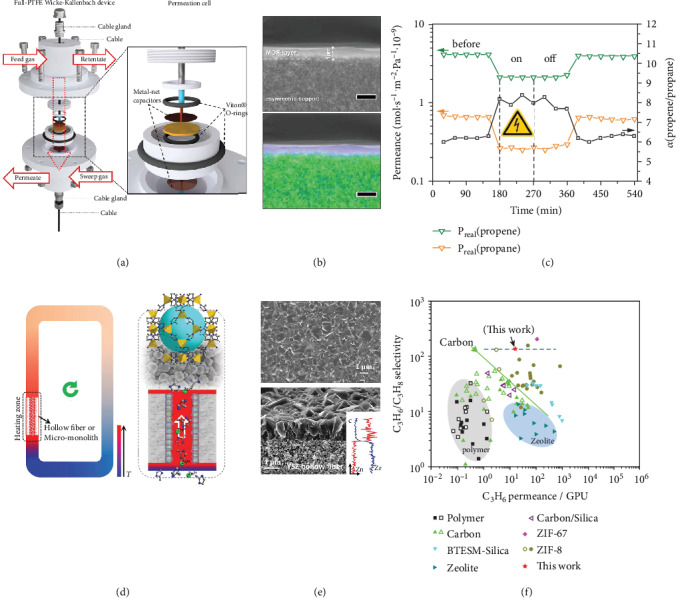
(a) The gas separation module coupled with electric field. (b) SEM images of the cross-section of ZIF-8 layer. Color code of EDXS (bottom): blue: Zn; green: Al. (c) Alkene-alkane separation performances of ZIF-8 membranes switched by electric field of 500 V/mm. Reproduced from Ref. [32] with permission from the American Association for the Advancement of Science, copyright 2017. (d) Illustration of the convective circulation synthesis loop. (e) SEM images of the surface (top) and cross-section (bottom) of a ZIF-8 membrane. (f) A wide comparison of membranes for C_3_H_6_/C_3_H_8_ separation. Reproduced from Ref. [35] with permission from Wiley, copyright 2018.

MOF-based membranes are suitable candidates for alkene/alkane separations. A rational design of grain boundaries and intrinsic pore structures of MOF membranes is required for optimum selectivity and permeability. Alkene-selective MOF membranes were reported to be derived from interfacial synthesis processes wherein two kinds of precursor solutions were divided by a porous substrate as a barrier, concomitant with the polymerization driven by counter-diffusion of solutions and triggered at the contact interface [27, 28]. Furthermore, heteroepitaxially grown MOF membranes were reported to exhibit unprecedentedly high propylene/propane selectivity as a result of the reinforcement of grain boundary structures [29, 30]. The intrinsic pore structures of MOF materials, like the dimension, size, orientation, and flexibility, are also key factors which determine the selective intracrystalline diffusion of gas molecules and separation performances of membranes. In recent years, researchers have been more aware of the flexibility effect of nanopores of MOFs on gas adsorption and separation characteristics. Porous MOF materials with the significant framework flexibility exhibited an induced-fit behavior for specific molecules via the pore structure adjustment [31]. Nevertheless, flexibility of MOFs is a two-edged sword. The pore opening, shear deformation, and other modes could result in undesirable separation performances. Knebel et al. proposed stiffening and defibrillation of soft ZIF-8 membranes with electric fields (Figures 4(a)–4(c)). The lattice polarization occurred when imposing an electric field (500 V/mm) to ZIF-8 membranes, and the crystal phase changed from standard cubic to polarized monoclinic and triclinic polymorphs [32]. The electric-switchable molecular sieving properties have been demonstrated in ZIF-8 membranes for propylene/propane separations. Knebel et al. predicted that higher E-fields could induce a full phase transformation that provided an improved molecular sieving capability. Recently, Zhou et al. reported that ZIF-8 membranes with the inborn-distorted and stiffer frameworks could be directly synthesized by a fast current-driven strategy, with polarized monoclinic polymorphs accounting for 60 to 70% of the membrane composition. Importantly, suppression of linker mobility in this new polymorph led to a sharpened molecular sieving capability for propylene/propane separations [33].

Alkene-selective MOF membranes have experienced substantial growth and breakthroughs during the past few years. An urgent demand has spurred researchers to explore easily scalable manufacturing technologies. Nair and coworkers innovated a method as an interfacial microfluidic membrane processing (IMMP) for manufacturing ZIF-8 membranes [34]. The metal-linker coordination reaction was confined into a microscopic space provided by a Torlon hollow fiber substrate. Interestingly, the modular approach permitted membrane fabrication and permeation simultaneously and, moreover, allowed membrane manufacturing in multiple fibers in parallel. Li and coworkers synthesized defect-free ZIF-8 membranes on the inner surface of ceramic hollow fibers by using a pump-free synthesis method in which hollow fibers were mounted into a closed loop of stainless steel tubing and heated to generate temperature differences to drive convection and circulation of the reactant solution (Figures 4(d)–4(f)) [35]. The membranes showed a remarkable propylene/propane separation performance, which demonstrated a facile, maintainable, and scalable method for alkene-selective MOF membranes. Some researchers were also dedicated to the fabrication of MOF membranes at low-cost and easily processable polymeric porous substrates. A great challenge is to promote nucleation on polymeric substrates and improve the binding strength between MOF layers and substrates [36].

### 2.4. Water-Selective Membranes

Dehydration by membrane pervaporation (or vapor permeation) is a promising energy-efficient technology for the refinery of organics. The core is materials with hydrophilicity and hydrothermal stability. Inborn water-stable MOFs mainly include metal-carboxylate frameworks with high-valence metal ions (e.g., Al^3+^-based CAU series, Zr^4+^-based UiO series, and MIL series) and metal-azolate frameworks with nitrogen-donor ligands (e.g., MAF series and ZIF series) [37]. Jin et al. prepared compact alumina supported hydrophilic CAU-10-H membranes by using secondary growth in a microwave oven, which could dehydrate ethanol [38]. Liu et al. synthesized UiO-66 polycrystalline membranes on the prestructured yttria-stabilized zirconia hollow fibers by an in situ solvothermal method. By modifying synthesis parameters (heating temperature, duration, and composition of solution), the well-intergrown membranes displayed exceptional performances for dewatering of butanol, furfural, and other organics [39].

MOF nanoparticles with interconnected nanopores for processing membranes via a mixed matrix strategy could improve the dehydration performance of hydrophilic polymer membranes [40–47]. Wu et al. synthesized hydrophilic UiO-66, UiO-66-OH, and UiO-66-(OH)_2_ nanoparticles by altering linkers and incorporated them into poly(vinyl alcohol) membranes. Pervaporation test revealed an anti-trade-off effect of MOF-based membranes for ethanol dehydration and that the swelling degree of MMMs declined by 28% in contrast to the pristine polymer membranes [48]. Chen et al. prepared Fe(III)-HMOF-5 with a hollow microstructure and incorporated them into a sodium alginate matrix membrane to achieve water permeation [49]. The coordinative vacancy of Fe^3+^ offered preferential interaction sites with water molecules, and the hollow structure of MOFs guaranteed the diffusion of water molecules, leading to a significant enhancement of dehydration performances. Li et al. reported that super-hydrophilic MOF-801 crystals consisting of Zr_6_O_4_(OH)_4_(CO_2_) secondary building units with exceptional water stability were introduced into a chitosan matrix [50]. An optimal performance was achieved due to pervaporation of membranes with 10 wt% water/ethanal solution at 343 K. Wang and coworkers modified ZIF-8 particles with ethane diamine and then doped them into PVA polymer membranes. Thanks to the improved hydrophilicity of ZIF-8 and strengthened interaction of ZIF with the polymer, the separation performance of hybrid membranes improved strikingly [51]. Zhang et al. coated hydrophilic SO_3_H-MIL-101-Cr by polydopamine (PD). The self-polymerization of the thin PD layer could prevent MOF particles from aggregation and be helpful to enhance compatibility between the filler and polymer matrix, thus providing an optimal excellent dehydration performance for ethylene glycol in the case of the MOF loading up to 30 wt% [52]. Wang et al. also authenticated the utility of ultrastable NH_2_-UiO-66 in acetic acid dehydration [53].

**Figure 5:**
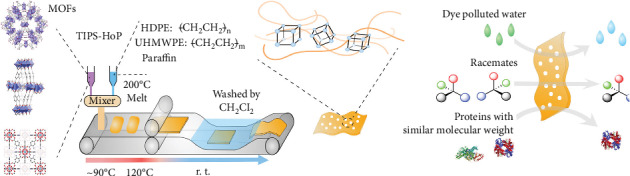
Illustration of the MOF-based MMMs synthesized by the TIPS-HoP method for separations. Reproduced from Ref. [61] with permission from Springer Nature, copyright 2019.

Water-selective membranes are of vital importance in water purification and desalination [54–60]. Wang et al. prepared diverse MOF-based MMMs with a hot-pressing (HoP) method coupled with a thermally induced phase separation (TIPS) technology (Figure 5) [61]. In this process, MOF nanocrystals are first homogenously mixed with the melt of high-density polyethylene (HDPE), ultrahigh-molecular weight polyethylene (UHMWPE) and paraffin at 200°C. Then, the mixture was poured onto the belt, followed by roll-to-roll hot pressing to form membranes at 120°C. The flexible membranes with ultrahigh MOF loading (approximately 86%) manifested a fast water permeation with rejection ratio of organic dyes reaching 99%. The facile approach showed potential for mass manufacture of MOF membranes for water purification.

### 2.5. Organic-Selective Membranes

Preferential permeation and recovery of organics using organic-selective membranes from the diluted aqueous solution is a complementary technology to dehydration of organics with excessive water. Accordingly, an integrated membrane separation technique might help; organic-selective membranes can be used for bulk separation and water-selective membranes for polishing the product.

The hydrophobicity of MOF materials is one of the most desirable properties besides the pore structure as well as the thermal and chemical stability, which determined permeation behaviors of target organic compounds in water-organic separation systems [62–64]. MMMs composed of these MOF nanoparticles embodied into the hydrophobic polymer are promising candidates for organic recovery from a diluted aqueous solution particularly from the fermentation broth [65–72]. Yang and coworkers incorporated ZIF-8 nanoparticles in silicone rubber (polymethylphenylsiloxane, PMPS) membranes to fabricate MMMs for recovering bioalcohols from a dilute aqueous solution (1.0 wt.%-3.0 wt.%) by pervaporation [73]. For example, the isobutanol permeance of the ZIF-8-PMPS membrane is 6000-7000 GPU (1 GPU = 10^−6^cm^3^ (STP) cm^−2^s^−1^cmHg^−1^), and the separation factor of isobutanol over H_2_O was above 40. The excellent organic-selective performances could be attributed to the hydrophobicity and flexible gate-opening effect of ZIF-8. The energy required for pervaporation using MMMs per unit of isobutanol is only half of that of distillation by simulation. By using a creative “plugging-filling” method and flexible mesh support with high porosity, the weight content of ZIF-8 nanoparticles was elevated above 40 wt%. And highly efficient recovery of furfural from water solution (1.0 wt%) was realized at 80°C [74]. By incorporation of hydrophobic substituted-imidazole linkers on the shell of ZIF-8 via ligand exchange, the hydrophobic and water-resistance of ZIF-8 further improved. Accordingly, the derived MMMs displayed improved organic selectivity [75].

In addition, considerable attention is being paid to achieve diverse liquid separation such as petrochemical separation and chiral resolution by using MOF-based membranes. Wu et al. fabricated MIL-160 membranes on polydopamine- (PDA-) modified *α*-Al_2_O_3_ disks via an in situ hydrothermal method and achieved p-xylene separation from o-xylene [76]. Kang et al. also demonstrated the feasibility of chiral MOF membranes for separations of chiral compounds. The Ni_2_(L-asp)_2_(bipy) membrane with neutral chiral Ni (L-asp) layers connected by 4,4′-bipyridine (bipy) linkers assembling in a pillared structure was directly synthesized on a nickel net that performed as a single nickel source. The separation result for a diol isomer mixture (2-methyl-2,4-pentanediol) revealed a preferential penetration of R diols through membranes and a high ee value, suggesting a geometry-dependent interaction between the chiral channel and R diols [77].

## 3. MOF-Based MRs: A Promising “Super Factory” Coupling Separations with Chemical Reactions

MOF-based MRs can be classified as (i) inert MRs wherein the catalyst bed is fixed at the membrane upstream or (ii) catalytic MRs wherein the catalyst is embedded into the membrane or the membrane itself has catalytic activity. Herein, we present MOF-based MRs applied in H_2_ production, dehydration, and organic extraction and illustrate their significance in intensified processes, with no emphasis on the function of the configuration of MRs.

### 3.1. H_2_ Production MRs

The current commercial technology for H_2_ production is to convert the fossil fuel to syngas, which is typically produced by gasification or reforming, such as steam-methane reforming (SMR) processes (500-900°C) or autothermal reforming (ATR) processes (500-900°C), followed by water-gas shift (WGS) processes (200-400°C) [78]. The syngas consists mainly of H_2_ and CO_2_ but also some unconverted or partly converted by-products like CH_4_, CO, H_2_S, and N_2_. H_2_-selective membranes as MRs are a feasible way to shift the intrinsic chemical equilibrium by virtue of selective permeation of H_2_. In particular, H_2_ production integrated with CO_2_ capture could be a key technology for simultaneously harvesting high-purity H_2_ and reducing CO_2_ emissions, which needs membranes with superior chemical stability and high permselectivity towards H_2_. The considerable hydrothermal and thermochemical stability is well-known for some MOFs including ZIF, MIL, and UiO microcrystalline powders in the temperature range of 200-400°C, which make MOF membranes available in WGS-MRs.

Yin et al. constructed low-temperature WGS-MRs based on well-intergrown ZIF-8 membranes with Cu/Zn/Al_2_O_3_ as catalysts. ZIF-8 WGS-MRs showed a remarked enhancement in the CO conversion (>13%) and H_2_ recovery. The performance of ZIF-8 MRs could be further optimized by modifying the operating temperature and space velocity to realize a “matching pot” between reaction kinetics and membrane permeation rates [79]. Lee et al. employed the composite substrate (MgO-Al_2_O_3_) to synthesize ZIF-7 membranes via the secondary growth method. The ZIF-7 membrane on the composite MgO-Al_2_O_3_ substrate displayed remarkably high thermal and hydrothermal stability by comparison with the ZIF-7 membrane on the bare *α*-Al_2_O_3_ support, since the latter provided a slight acid substrate surface causing the break of Zn-N bonds and decomposition of the ZIF-7 framework while the former offered acid-base neutrality at the membrane-substrate interface to stabilize ZIF-7 [80]. The gas permeation stability of the ZIF-7 membrane on the composite MgO-Al_2_O_3_ substrate was also authenticated by exposure of the membrane to a gas mixture composed of H_2_, CO, CH_4_, and CO_2_ at harsh conditions (200-400°C, 0-40% H_2_O) for approximately 100 h, whereas the permselectivity of the membrane on the bare Al_2_O_3_ substrate declined rapidly in a relatively mild condition (300°C, 20% H_2_O) because of the partial collapse of the ZIF-7 framework. ZIF-7/MgO-Al_2_O_3_ membranes such as WGS-MRs led to a significant enhancement in CO conversion and H_2_ recovery relative to a conventional reactor. The H_2_ recovery at the downstream was above 50%, and the concentration of CO and CO_2_ was only 0.2% and 4.2% in the stream, respectively, yielding a potential opportunity of MOF-based WGS-MRs for clean H_2_ production.

A further improvement in the thermal and hydrothermal stability of MOF membranes is needed for industrial application of MOF-based WGS-MRs. Additionally, MOF-based MRs would be extended to diverse H_2_ production processes such as H_2_ production from ammonia and other renewable sources. In the future, MOF-based MRs would be potentially applied to intensify some emerging H_2_ production processes, such as clean and sustainable electrocatalysis and photocatalysis routes.

### 3.2. Dehydration MRs

Water as a by-product emerges in several catalytic conversions, for example, the reactions producing chemicals (e.g., acetic acid and esters) [81–84] or fuels (e.g., methanol, dimethyl ether, and linear paraffins) [85–87]. The water sometimes poisons the catalyst and may inhibit the catalytic reaction owing to thermodynamic limitations. The in situ water removal with MRs is a promising solution, leading to strengthened efficiency and saved costs.

Esterification reactions in dehydration MRs are typical intensified processes [88]. Sorribas and coworkers synthesized MMMs based on the polyimide with hydrophilic HKUST-1 as porous nanostructured fillers and applied them in dehydration MRs for esterification reactions [89]. The membrane was fixed in a stainless-steel permeation module, and the equimolar acetic acid-ethanol mixture with constant flow rate was fed. The esterification reaction was carried out in this module with the ionic exchange resin Amberlyst 15® as a catalyst, concomitant with in situ dewatering by membrane pervaporation. As a result, the conversion in MRs improved remarkably. An excellent dehydration capacity of MRs was also demonstrated during the first 10 h, corresponding to the water content in the permeant reaching around 90%. Nevertheless, the membrane was ultimately damaged under an acidic reaction medium after 30 h, and the water molar fraction in the permeant decreased to 69%. To tackle this problem, researchers synthesized MMMs based on MIL-101 (Cr) nanofillers that have an excellent resistance to the harsh reaction medium [90]. The results suggested that the presence of MIL fillers improved the membrane permeation flux and led to higher conversions. Importantly, MIL-based MRs exhibited constant conversion and permeation flux for more than 3 days.

Currently, MOF-based dehydration MRs are mainly employed in esterification systems. MMMs were the most popular membranes. For avoiding membrane damage by the acidic liquid system, another feasible way is to employ vapor permeation instead of pervaporation. Furthermore, pursuit of intensified processes for producing high added-value chemicals would motivate the development of MOF-based dehydration MRs compatible in diverse chemical reactions (e.g., the Knoevenagel reaction) that generate excessive amount of water by-products. Moreover, ZIF-8 and other MOFs with amine-decorated linkers have been reported to be an efficient heterogeneous catalyst for the low-temperature liquid phase Knoevenagel reaction [91]. Therefore, the design and synthesis of catalytic ZIF-8 MRs or other MOF MRs would be a direction in the future, in which the membrane itself possesses both catalytic activity and molecule permselectivity.

### 3.3. Organic Extraction MRs

**Figure 6:**
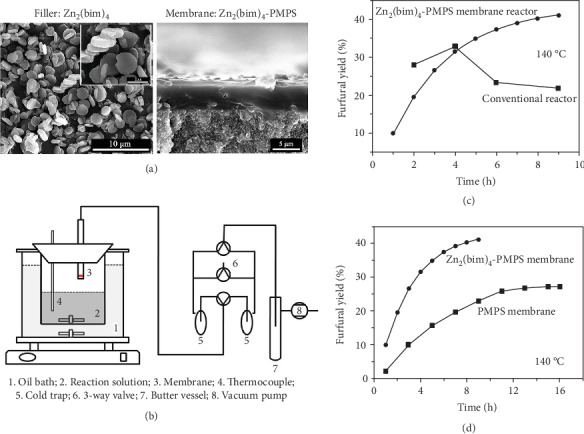
(a) SEM images of Zn_2_(Bim)_4_ fillers and MMMs. (b) Experimental set-up of the membrane reactor for conversion of xylose. (c, d) Furfural yield achieved in the Zn_2_(Bim)_4_-PMPS MR compared with that in a conventional reactor and a PMPS MR. Reproduced from Ref. [92] with permission from Elsevier, copyright 2016.

Organic extraction MRs are innovative processes for in situ recovery of high purity target organic compounds during production. Yang and coworkers demonstrated the utility of MRs in the biomass-related conversion. The hydrophobic MMM comprising silicone rubber (polymethylphenylsiloxane, PMPS) and Zn_2_(Bim)_4_ (layered ZIF material) was employed for the in situ furfural recovery by vapor permeation integrated with xylose dehydration to achieve furfural (Figure 6) [92]. The furfural yield in MRs reached 41.1% after 9 h at 140°C, superior to the conventional reactor and the PMPS MR at the same condition. In addition to shifting the chemical equilibrium, another importance of furfural extraction MRs was to avoid undesirable side reactions, such as condensation, resinification, or decomposition.

The use of MRs in a nonaqueous system is an emerging area [93–95]. Bai et al. employed a facile spray-assisted miscible liquid-liquid interface (MLLI) method to synthesize CuBDC membranes, which performed as a flowing membrane reactor in the 4-nitrophenol reduction reaction [94]. Zhang and coworkers presented a catalytic NH_2_-MIL-53 membrane that generated in the channels of an anodized aluminum oxide (AAO) membrane [95]. The Knoevenagel condensation reaction over the membrane showed a high yield of around 50%. In the catalytic MRs, the products can also be removed fast from the reaction zone, which guaranteed the activity and stability during the long-term catalytic reaction and recycling process.

## 4. Brief Discussion on MOF Stability

We summarized the recent advances that render MOF materials as efficient separation membranes and MRs in diversified processes of industrial interest. The fascinating potential of MOF MRs motivated the design and synthesis of MOF materials with multifaceted networks, functionalities, and, more importantly, chemical/thermal/hydrothermal stability to withstand harsh chemical environments and thermally challenging conditions in the real industrial processes. The stability of MOFs seems to be a daunting and formidable issue in the conventional wisdom, since the early reported MOF materials encompassing divalent Zn^2+^ or Cu^2+^ and bidentate carboxylate ligands (e.g., MOF-5) have proven unstable in moisture and aggressive conditions. Recent years have witnessed the surge of numbers of MOF structures with the development of computational de novo design together with crystal engineering. Many stable MOFs have emerged. Previous reviews enclosed an exhaustive summary of MOFs that maintained crystallinity and porosity under high temperature (>400°C), an aqueous solution with a broad range of pH, or hydrothermal steaming environments, as exemplified with ZIFs, MAFs, MILs, and UiOs [37, 96, 97]. We emphasized four points for improvement of chemical/thermal/hydrothermal stability of MOF materials, referring to (i) combination of high valent metal nodes, such as Al^3+^, Zr^4+^, and Ti^4+^ with oxyanion-terminated linkers to enhance the metal-linker bond strength essentially; (ii) use of an inert metal node or linkers that can survive in oxidizing atmospheres or other forcing conditions; (iii) decoration hydrophobic linkers as substituents or positioning of a hydrophobic protective layer to shield MOFs from water/vapor aggression; and (iv) rational catenation or other densification approaches. Additionally, elimination of membrane cracks and reinforcement of membrane-support interface is conducive to further improve stability of MOF membranes. Undoubtedly, the urgent demand for more stable MOF membranes in MRs will further promote the on-purpose material design and membrane configuration control in the future.

## 5. Conclusion and Outlook

The significance of MOF membranes manifests in diverse separation fields, exemplified with H_2_ separation, CO_2_ capture, alkene/alkane separation, and organic dehydration and extraction. However, MOF membranes are still in lab-scale applications today. For achieving industrial applications of MOF membranes, two issues should be addressed in the near future, namely, (i) development of membrane materials with adequate permeability, selectivity, stability, and acceptable cost and (ii) exploration of facile, reliable, and scalable membrane fabrication strategies. Meanwhile, it is vital to consider the separation performances of MOF membranes in real-working conditions, including temperature, pressure, and humidity, and evaluate the role of trace contaminants.

The pursuit of economics and sustainability in modern energy consumptions and chemical manufacturing requires MRs to realize intensified processes. One huge challenge is to establish a well-matched relationship between chemical reaction kinetics and membrane permeation rates. MOF-based MRs would potentially be extended to CO_2_ conversion and hydrocarbon-related transformation in the future. An innovative concept is the design of CO_2_ conversion MRs by integrating CO_2_-selective MOF membranes with semiconductors for the photocatalytic reduction of CO_2_ into C_1_ building blocks to make organic chemicals, materials, and carbohydrates, which not only contributes to alleviating global warming but also provides opportunities in chemical industries [98]. MOFs have recently emerged as multifunctional heterogeneous catalysts [99]. Accordingly, an attractive frontier is the design of catalytic MOF MRs in which the MOF layer possesses both catalytic activity and separation capacity. Although MOF MRs are a relatively new field, the efforts to exploit their potential will grow considerably in the future.
